# Wide-Band High Concentration-Ratio Volume-Holographic Grating for Solar Concentration

**DOI:** 10.3390/s20216080

**Published:** 2020-10-26

**Authors:** Chengchen Wang, Jianshe Ma, Hongxu Kao, Taihui Wu, Ping Su

**Affiliations:** Tsinghua Shenzhen International Graduate School, Tsinghua University, Shenzhen 518055, China; chengche19@mails.tsinghua.edu.cn (C.W.); ma.jianshe@sz.tsinghua.edu.cn (J.M.); khx17@tsinghua.org.cn (H.K.); taihuiwu@163.com (T.W.)

**Keywords:** solar concentration, volume hologram, photopolymer, wide band

## Abstract

Efficient and low-cost solar-energy collection has become the focus of many research works. This paper proposes a recording method and an experimental verification of a wide-band, large-angle, and high concentration-ratio volume-holographic grating for solar concentration. We applied the Kogelnik coupled-wave theory and photopolymer diffusion model to analyse the formation mechanism and influencing factors on the diffraction efficiency of monochromatic volume-holographic gratings. We design and construct a three-color laser-interference system to record three monochromatic volume-holographic gratings. The best recording conditions are determined by experiment and simulation. A trichromatic volume-holographic grating is obtained by gluing the three monochromatic gratings together. The experimental results show that the trichromatic volume-holographic grating with a working angle of 6.7° and a working band of visible light has a light concentration ratio of 149.2 under an illumination of the combined recorded three-color beams, and that under sunlight is 27.2. We find that the proposed trichromatic volume-holographic grating for light concentration offers the advantages of wide band and high light concentration ratio, which provide a reference for solar concentration.

## 1. Introduction

Non-renewable energy sources, such as coal, petroleum, and natural gas, not only cause environmental pollution, but also restrict economic development to a certain extent. Solving the energy problem has become an urgent issue to address in our contemporary society. As a type of renewable energy, solar energy provides the advantages of universality, safety, and long-lasting properties; therefore, it offers special development prospects. At present, traditional concentrators, such as lenses and Fresnel lenses, are widely applied, but they have small condensing angle. Hence, a tracking device must be installed when they are used as sunlight concentrator, which leads to issues such as complicated concentrator structure and high cost. Following the development of material technology, concentrators based on volume holography are expected to solve the small working-angle problem of traditional concentrators.

Ludamn et al. [1] first proposed holographic concentrators. Thereafter, the theory and method of holographic concentrators have been gradually developed. According to previous studies, many researchers studied the feasibility of solar concentrators and proposed various volume-holographic concentration structures [2,3,4,5,6,7,8,9,10,11,12,13,14,15,16,17,18,19,20,21,22]. Ren et al. proposed a three-layer volume-holographic tandem concentrator system [3] that could achieve a condensing angle of more than 60°. Akbari et al. proposed a four-layer volume-holographic concentrator system [11] that could concentrate light from 24°, and the monochromatic light concentration ratio was 20. Lee et al. proposed an angle-multiplexing volume-holographic concentrator system [18]. In their concentrator system, the object light was a spherical wave generated by a condensing lens, and the reference light was multiplexed three plane waves in which the angle of the three plane-wave beams was 10° apart. The diffraction efficiencies of the different angle ranges were 16.6%, 21.8%, and 26.5%. Although these systems could achieve a certain light-gathering effect, they had low light-gathering efficiency. Because of monochromatic holography, the dispersion effect was inevitable.

To improve sunlight utilisation, large-angle and multi-wavelength volume-holographic concentrator systems were proposed. Multi-layer volume-holographic solar-light concentrating structures [1,2,5], which consisted of the combination of three or more volume-holographic elements to achieve a large working angle and a wide-band light-gathering effect, were proposed. Ludman et al. [1] proposed a multi-layer concentrating structure in which each layer was divided into three areas to converge sunlight at different angles, which achieved a theoretical concentration angle of 100°; however, the solar concentration ratio is only 4.0. Stojanoff et al. [2] proposed a multi-layer light-concentrating structure where each layer had a different working angle. The simulation demonstrated that it could work in the wavelength range of 300–1500 nm with a working angle of 22°–80°. Although only theoretical, the proposal of this structure promoted the development of volume-holographic concentration. A multi-layer concentration structure proposed by Hung et al. [5] provided a concentration ratio of approximately 1.5 and efficiency of 50%. The above-mentioned structures aim to achieve light-gathering effect through a multi-layer structure; however, most of them suffer from problems such as low light-gathering ratio and large inter-layer interference.

Some works used other materials or concentrator structure, in combination with volume-holographic gratings [6,8,10], to obtain wide-band and large-angle light-gathering effects. Castillo et al. [6] proposed a single-layer volume-holographic grating sub-band light-collection system that used double-sided photovoltaic (PV) cells to reduce the loss caused by dispersion. Iurevych et al. [8] proposed a solar-energy collection device using polycarbonate plates combined with volume-holographic gratings. Part of the incident sunlight was diffracted by the holographic reflector and reached the PV cell through the total reflection in the polycarbonate plate. The other sunlight part was transmitted to the polycarbonate plate and reached the other PV cells. Gordon et al. [10] proposed a single-layer volume-holographic sub-band concentration system that recorded the different angles and wavelengths at the same location. Their system used the dispersion of volume-holographic materials to improve the light-energy utilisation efficiency of the overall system. The simulated system efficiency was 31.0%, which was an improvement by 11.9% over the best single PV cell. These systems realised volume-holographic light that focused on large angle and multi-wavelength. However, problems occurred, such as low concentration ratio, complex structure, and large interlayer crosstalk.

A high-efficiency volume-holographic concentrator requires the following factors [16,17,19]: (I) high-performance recording material, (II) large concentration ratio, (III) large angular selectivity, and (IV) utilisation of the entire solar spectrum. None of the aforementioned studies have comprehensively considered these four factors. Inspired by the previous work, we use the Kogelnik coupled-wave theory [20,21,23] and the first-order monomer diffusion model proposed by Piazzolla et al. [24] to achieve comprehensive consideration of the four factors and design and fabricate a volume-holographic concentrator, which possesses the characteristics of wide band, large angle, and high diffraction efficiency. For recording volume holographic gratings with high spatial frequency, the non-local diffusion model is recommended because a non-local material response function is introduced [25].

The rest of the paper is organised as follows. In Section 2, the design theory and method are introduced. In Section 3, the recording conditions and construction of the experimental platform are presented. In Section 4, testing of the optical characteristics of the system is discussed, and the results are compared with those of the simulation. In Section 5, the results and future trends are presented.

## 2. Design Theory and Method

According to previous studies, the present work starts from the Kogelnik coupled-wave theory [23] and establishes a volume-holographic solar-light concentration model.

**Figure 1:**
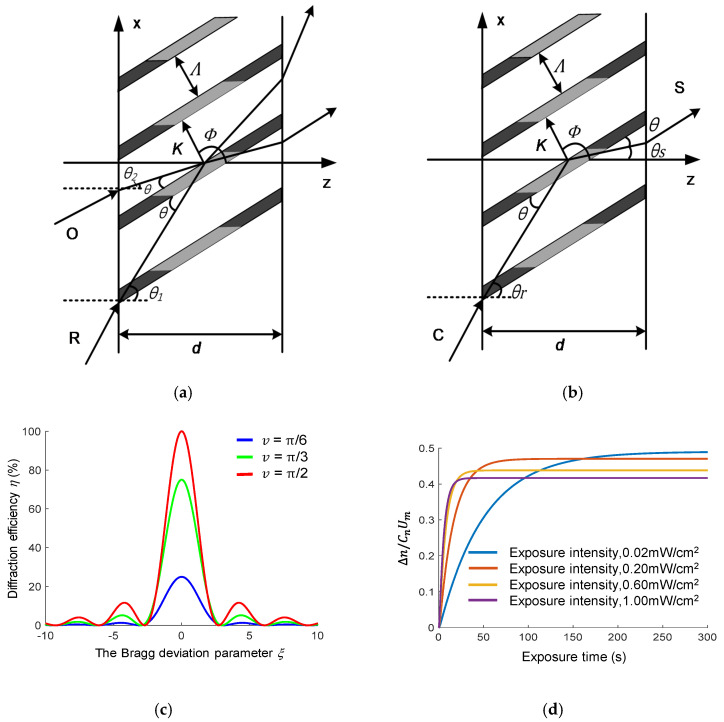
Sketches of (**a**) interference recording and (**b**) diffracted reconstruction of a volume-holographic grating. (**c**) Diffraction efficiency of the volume-holographic gratings, which varies with Bragg deviation parameter ξ under different coupling intensities. (**d**) The relationship between refractive index modulation and exposure intensity.

Figure 1a shows that two light beams interfere in a medium with thickness d, forming periodic light and dark interference fringes. The volume-holographic grating reproduces the object beam when illuminated by a beam, which is similar to the reference beam. When the angle of incidence, wavelength, and thickness of the medium satisfy the Bragg conditions, Bragg diffraction occurs, and the diffraction efficiency reaches the maximum. Equation (1) expresses the Bragg condition.

(1) 2Λsinθ=λ,

where θ is the angle between the two beams that satisfies the Bragg condition (or Bragg angle, for brevity), Λ is the fringe pitch of the volume-holographic grating (also called the grating constant), and λ is the wavelength of the incident-light wave in the volume-holographic medium.

When the incident-light angle or wavelength deviates from the Bragg angle and Bragg wavelength, the diffraction efficiency decreases, which illustrates the wavelength and angle selectivity of the volume holography. To achieve maximum diffraction efficiency, we use the approximate coupled-wave theory to find the appropriate wavelength and incident angle. Currently, the Kogelnik coupled-wave theory [23] is the most widely used theory for analysing the efficiency of the volume-hologram diffraction. In this theory, volume holography is considered to be the exchange in energy between the diffracted and incident waves. The diffraction efficiency is defined as the ratio of the two intensities. According to the Kogelnik coupled-wave theory, the diffraction efficiency is expressed as [23]

(2) $$\eta_{T}=\frac{\sin^{2}{\left(v^{2}+\xi^{2}\right)}^{1/2}}{1+\xi^{2}/v^{2}}$$

where parameter $\eta_{T}\;$is the diffraction efficiency of the volume hologram, $\xi =\Delta \theta kd\sin(\phi -\theta_{0})/2q_{s}-\Delta \lambda k^{2}d/8\pi nq_{s}$ is the Bragg deviation parameter of the incident reference or object light wave, and v=πΔnd/λcosθ is the coupling strength between the object and reference light waves in the volume hologram, where Δ*n* can be obtained by a diffusion model.

Figure 1c shows the diffraction-efficiency curve that varies with Bragg deviation parameter *ξ* when *ν* is equal to π/6, π/3 , and π/2 within a period. We can observe that coupling intensity ν affects not only the gradient but also the peak of the diffraction efficiency. Therefore, after the material selection, the best diffraction efficiency can be obtained by studying the wavelength and angle selectivity.

According to the thickness of the holographic recording medium, the recorded holograms can be classified into plane and volume holograms, also known as surface and thick holograms, respectively. Distinguished variable *Q* is expressed as [26]

(3) $$Q=\frac{2\pi \lambda_{s}d}{n\Lambda^{2}}$$

where $\lambda_{s}$ is the incident-light wavelength in a medium with refractive index n, which is related to wavelength $\lambda_{0}$ of light in the vacuum according to $\lambda_{s}=\lambda_{0}/n$. d is the thickness of the holographic material substrate. When *Q* ≥ 10 the recorded hologram is a volume hologram; otherwise, it is a plane hologram.

We employ the first-order monomer diffusion model proposed by Piazzolla et al. [24] to quantitatively describe the influence of each parameter on the recording process. The analytical formula for the refractive-index modulation is expressed as follows:

(4) $$\Delta n(t)=\frac{m\tau \Delta n_{M}}{\tau_{D}+\tau }\left\{1-\exp\left\{\gamma \left[1-\exp\left(\frac{t}{\tau }\right)\right]\right\}-\frac{\tau_{D}\gamma }{\tau_{D}\gamma +\tau }\left[1-\exp(-\frac{\tau_{D}\gamma +\tau }{\tau_{D}\gamma }t)\right]\right\}$$

where $\Delta n_{M}=C_{n}\delta U_{m}$ represents the maximum amplitude of the grating modulation. $U_{m}$ is the initial monomer concentration, and $C_{n}$, m, and γ are constants. $k_{0}$ represents the polymerisation coefficient. $\tau =1/\varphi I^{\delta }$ denotes the polymerisation time constant. $\tau_{D}$ is the diffusion time constant. Parameters δ and φ are constants whose values are related to the material, and I is the exposure intensity. It can be seen from v=πΔnd/λcosθ that the coupling strength v is related to the refractive index modulation degree, and from Equation (4), the refractive index modulation degree is related to the exposure intensity, the polymerization coefficient and the diffusion time constant. Because polymerization coefficient and diffusion time constant are unique properties of polymers, the degree of refractive index modulation can be changed by changing the exposure intensity. After the relevant material parameters are determined, the relationship curve between refractive index modulation and exposure intensity is shown in Figure 1d. When the exposure intensity is high, the saturation refractive index modulation decreases; when the exposure intensity is small, the saturation refractive index modulation of the polymer increases.

**Figure 2:**
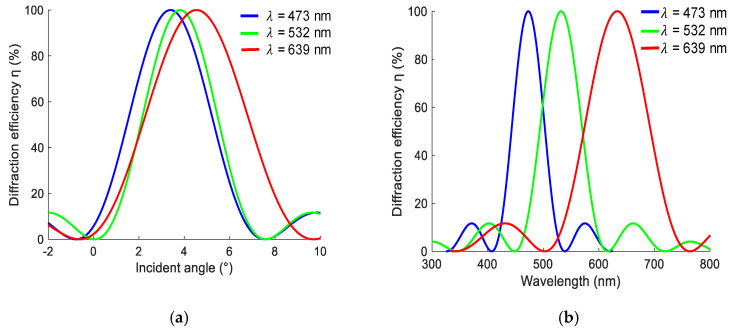
(**a**) Angular and (**b**) wavelength selectivity values of red green and blue colours.

In this study, Bayfol HX200 [27,28,29] is used as the volume-holographic recording material, whose thickness is 16 ± 2 μm. The average refractive index at 532-nm wavelength is 1.5. The sensitive wavelength of the material covers the range from 400 to 700 nm, which is suitable for solar-light concentration. To record a volume hologram, we choose *Q* as 24. By substituting the incident wavelength into Equation (3), the spatial frequencies of the gratings under the 639 nm red light, 532 nm green light, and 473 nm blue light are $S_{R}=$ 483.3 lp/mm, $S_{G}=$ 529.7 lp/mm, and $S_{B}=$ 561.8 lp/mm, respectively. The angular and wavelength selectivity values of the three volume-holographic gratings are shown in Figure 2.

Figure 2a shows that the green volume-holographic grating demonstrates the highest angular selectivity, and the red volume-holographic grating exhibits the lowest angular selectivity. Figure 2b shows that the blue volume-holographic grating exhibits the highest wavelength selectivity, whereas the red holographic grating exhibits the lowest wavelength selectivity. Therefore, the working angle of the wide-band volume-holography system should be based on the angle of the blue light. The volume-holographic diffraction efficiency based on photopolymer materials can reach a maximum of 81.9% under a 639 nm red light, 82.7% under a 532 nm green light, and 82.3% under a 473 nm blue light. In terms of the working angle, the working angles obtained at 473, 532 and 639 nm are 6.7°, 7.5°, and 9.0°, respectively. Its working band covers the entire visible-light spectrum.

## 3. Recording and Concentration Performance of The Volume-Holographic Gratings

**Figure 3:**
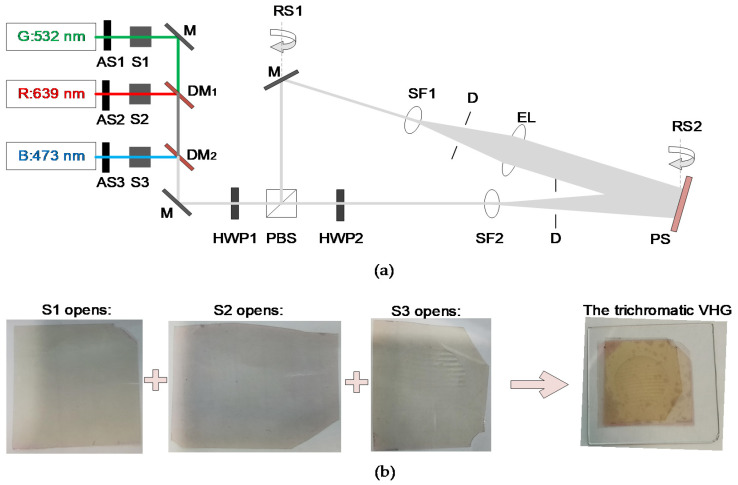
(**a**) Schematic diagram of the RGB three-color interference light path. (**b**) Schematic diagram of the three-color condenser formation. AS: continuously adjustable attenuator, S: controllable shutter, M: mirror, DM: dichroic mirror, HWP: half-wave plate, PBS: polarisation beam splitter, SF: spatial light filter, D: aperture. EL: beam expander, PS: photopolymer stage, and RS: rotation stage.

We design and construct a laser-interference system using 473-, 532-, and 639 nm illumination wavelengths to record the wide-band volume-holographic grating. The schematic diagram and photograph of the experimental platform are shown in Figure 3a,b.

The models of the three solid-state lasers from top to bottom are MSL-R-532, MSL-FN-639, and MSL-FN-473 (Changchun New Industry). The output power of the three lasers is relatively large. Thus, three continuously adjustable attenuators (ASs) are installed after the three lasers, and three optical shutters (Ss), which are controlled by a timer, are installed behind the attenuators to control both the exposure time and illumination colour. In this trichromatic light path, two dichroic mirrors (DMs) are installed to combine the beams with different colours.

The wide-band volume-holographic grating is obtained according to the following process. First, shutter 1 (S1) is opened for certain seconds, and a red volume-holographic grating is recorded. Second, a piece of photopolymer is changed, shutter 2 (S2) is opened for certain seconds, and a green volume-holographic grating is recorded. Third, another piece of photopolymer is changed, shutter 3 (S3) is opened for certain seconds, and a blue volume-holographic grating is recorded. Each hologram is cured for some time using a mercury lamp after the recording. Finally, the three monochromatic volume holograms are manually aligned and glued together, and the wide-band volume hologram is obtained. Figure 3b shows the schematic of the recording procedure.

Volume-holographic gratings are formed because the refractive index of the substrate is modulated by the interference fringes, and diffusion and polymerisation of monomers subsequently occur in the substrate. The degree of refractive-index modulation is positively correlated with the diffraction efficiency. The parameters in the exposure and the curing affect the degree of refractive-index modulation. We experimentally studied the effect of exposure energy and the ratio between the object-beam and reference-beam energy on the diffraction efficiency of the volume-holographic grating.

**Figure 4:**
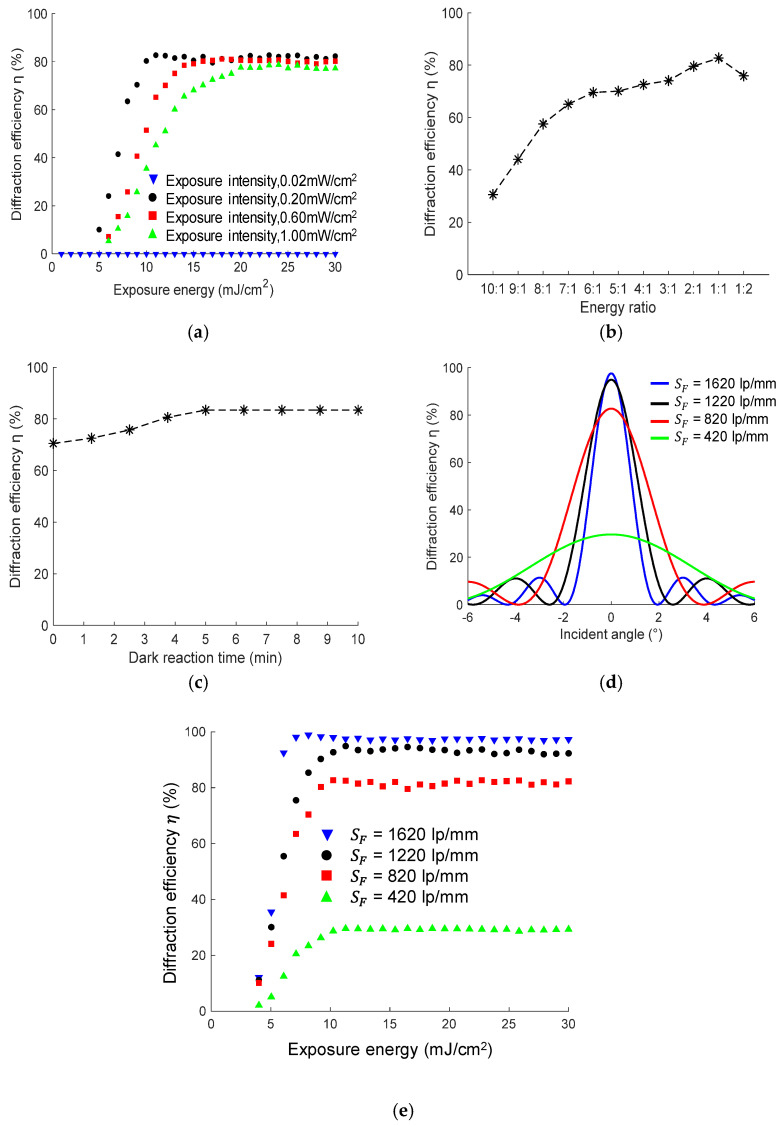
Influence of some processing parameters on the diffraction efficiency of the volume-holographic grating: (**a**) exposure intensity and exposure energy, (**b**) ratio of the object to the reference energy, (**c**) dark reaction time according to experiments, (**d**) different working-angle ranges of the volume-holographic gratings recorded under different spatial frequencies, and (**e**) diffraction efficiency of volume holographic gratings with different spatial frequencies under different exposure energies by experiment.

By adjusting the output power of the laser controller and rotating HWP1, the exposure intensity—which is measured using an optical power meter—can change. We investigated the situations when the exposure intensities are equal to 0.02, 0.2, 0.6, and 1.0 mW/cm^2^. Under a certain exposure intensity, we set different exposure times to adjust the exposure energy to 0–30 mJ/cm^2^. A total of 120 volume-holographic gratings under an illumination of 532 nm laser were recorded and cured under the same condition, and their diffraction efficiency values were measured; the result is shown in Figure 4a.

The ratio between the object-beam and reference-beam energy is adjusted by rotating HWP1, from 10:1 to 1:2. Different volume-holographic gratings illuminated by 532 nm laser were recorded and cured under different energy ratios. The relationship between the diffraction efficiency of the volume-holographic gratings and energy ratio is shown in Figure 4b, which shows that when the ratio of the light intensity of the object and reference light paths gradually decreases, the diffraction efficiency value of the volume hologram gradually increases. The diffraction efficiency reaches the maximum when the light-intensity ratio of the two beam paths is 1:1.

The dark reaction time, which is the time between the recording and curing, can also affect the diffraction efficiency. We change the dark reaction time in the production procedure and study the effect of the dark reaction time on the diffraction efficiency; the result is shown in Figure 4c. When the dark reaction time is zero, the curing process is directly performed after the interference exposure. Under this situation, the volume-hologram diffraction efficiency is approximately 70%. When the dark reaction time increases, the diffraction efficiency gradually increases. When the dark reaction time is approximately 5 min, the diffraction efficiency of the polymer material reaches the maximum value and remains stable with the continuous increase in the dark reaction time, which indicates that the refractive-index modulation is saturated.

The spatial frequency, which is determined by the angle between the object and reference beams, also affect the diffraction efficiency of the volume-holographic grating. We use Equation (2) to calculate the diffraction efficiency of the volume-holographic grating under different monochromatic incident angles, and the volume-holographic grating is recorded according to the angles between the object and reference beams corresponding to the spatial frequencies of 420, 820, 1220, and 1620. The results are shown in Figure 4d. By rotating RS1 and adjusting the follow-up optical path, different spatial frequencies can be obtained and the diffraction efficiency of the recorded holographic gratings are tested under different exposure energies, the result of which is shown in Figure 4e. The measured maximum diffraction efficiency of volume holographic gratings with four different spatial frequencies are in accordance with the simulated results in Figure 4d.

Figure 4a shows the experimental results under different exposure intensities on the diffraction efficiency. The selected exposure intensities are 0.02, 0.2, 0.6, and 1.0 mW/cm^2^. We can observe that the change trend in the diffraction efficiency is consistent with the simulation results. Higher diffraction efficiency can be achieved at lower exposure intensity. However, with the increase in the exposure intensity, the diffraction efficiency of the volume hologram becomes the largest. The value is reduced; thus, the diffraction efficiency obtained through the experiment is 0.2 mW/cm^2^, and the diffraction efficiency becomes the best. At this time, the maximum diffraction efficiency value is 82.7%. Subsequent experiments are conducted at this exposure intensity.

Our result reveals that the larger the spatial frequency is, the larger is the diffraction efficiency of the volume hologram. When the spatial frequency is 820 lp/mm, the maximum diffraction efficiency is 82.7%, and the working angle is 7.5°. As the spatial frequency increases, although the diffraction efficiency also increases, the working-angle range becomes significantly small. Therefore, a larger spatial frequency is not conducive for the application of volume-holographic gratings for the condensation of a solar-concentration system. In our case, we choose a spatial-frequency value of 820 lp/mm, which corresponds to an intersection angle of 7.5° between the reference and object beams. We adjust the angle using a manual rotation stage.

According to the aforementioned discussions, to obtain volume-holographic gratings with high diffraction efficiency, the exposure intensity under a uniform light intensity is 0.2 mW/cm^2^, that is, the exposure intensities of the reference-light and object-light paths are both 0.1 mW/cm^2^, and the angle between the two beam paths is adjusted to 7.5° so that the spatial frequency is 820 lp/mm. The three monochromatic volume-holographic gratings are recorded at the same grating spatial frequency; thus, the angle between the two beam paths remains unchanged. The dark reaction time is 5 min. The working angle of the trichromatic volume-holographic grating is 6.7°, which is equal to that of the blue volume-holographic grating. The working band is the visible band, that is, the trichromatic volume-hologram diffraction reproduces the concentrated sunlight.

## 4. Results

To characterise the optical properties of the monochromatic volume-holographic gratings, we illuminate them using the corresponding three monochromatic lasers used for the recording. To characterise the optical properties of the cohesive trichromatic volume-holographic grating, we illuminate it using the combined beam of the three monochromatic lasers used for the recording and using sunlight.

### 4.1. Optical Properties of The Monochromatic Volume-Holographic Gratings Illuminated by The Corresponding Three Recording Lasers

**Figure 5:**
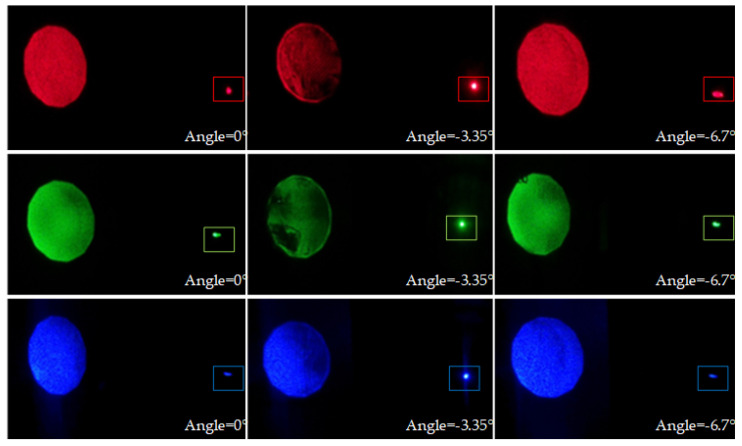
Light distributions on the focal plane of the three monochromatic volume-holographic grating illuminated by the three recording collimated lasers at 0°, −3.35°, and −6.7° angles; the spots in the squares represent the focal points.

Figure 5 shows the test results of the three monochromatic volume-holographic gratings under the illumination of the corresponding recording monochromatic collimated lasers and with incident angles of 0°, −3.35°, and −6.7°. In each sub-figure, the big light spot at the left side denotes the transmitted light, and the small light spot at the right side denotes the diffracted converging light spot. By rotating the rotating stage that holds the monochromatic volume-holographic grating, the diffraction effect at different incident angles can be observed. The −3.35° angle represents the blue volume-holographic grating Bragg angle, and under this condition, the green and red monochromatic volume-holographic gratings work near the Bragg condition. Thus, they also have high diffraction efficiency. The other two angles deviate quite far away from the Bragg angle. Thus, under these conditions, the volume-holographic gratings have quite a low diffraction efficiency.

### 4.2. Optical Properties of The Trichromatic Volume-Holographic Grating Illuminated by The Combined Beam of The Three Recording Lasers

**Figure 6:**
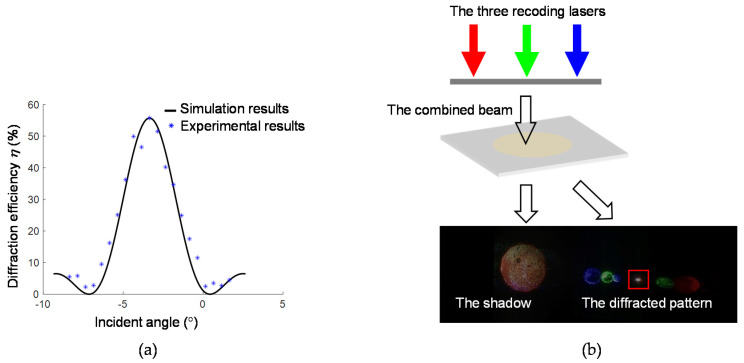
(**a**) Relationship between the working angle and diffraction efficiency of the trichromatic volume-holographic grating. (**b**) Light distribution on the focal plane of the trichromatic volume-holographic grating at an incident angle of −3.35° illuminated by the combined three laser beams. Inside the red square is the focal spot.

The trichromatic volume-holographic grating is illuminated by the mixed collimated light from the three recording lasers, which is obtained by turning on all the three shutters in the recording optical path. By rotating the rotation stage that holds the trichromatic volume-holographic grating, the diffraction effect at different incident angles can be observed, and the diffraction efficiency can be measured using an optical power meter. The result is shown in Figure 6a. Figure 6a also shows the simulated relationship between the diffraction efficiency and incident angle, which is quite consistent with the experiment result. It also shows that the working angle of the single-layer RGB volume-holographic grating is 6.7°. At an incident angle of −3.35°, the diffraction efficiency reaches the maximum value of 55.7%. The light distribution on the focal plane at the incident angle of −3.35° is captured and shown in Figure 6b. The light spot next to the focus point in Figure 6b is caused by the chromatic dispersion of monochromatic volume holographic gratings. A charge-coupled device (MV-EM510M; pixel pitch = 3.45 μm) is used to image the focal spot and measure its size. The focal spot occupies approximately 408 pixels on the sensor plane, which corresponds to a diameter of 0.704 mm. The geometric focusing ratio is calculated using the ratio of the focal-spot area to the incident-beam area, i.e., $Ρ=S_{0}/\;S_{0}^{\prime }=149.2$. The average light energy utilisation rate in this angle range is 26.54%.

### 4.3. Optical Characteristics of The Trichromatic Volume-Holographic Grating Illuminated by Sunlight

**Figure 7:**
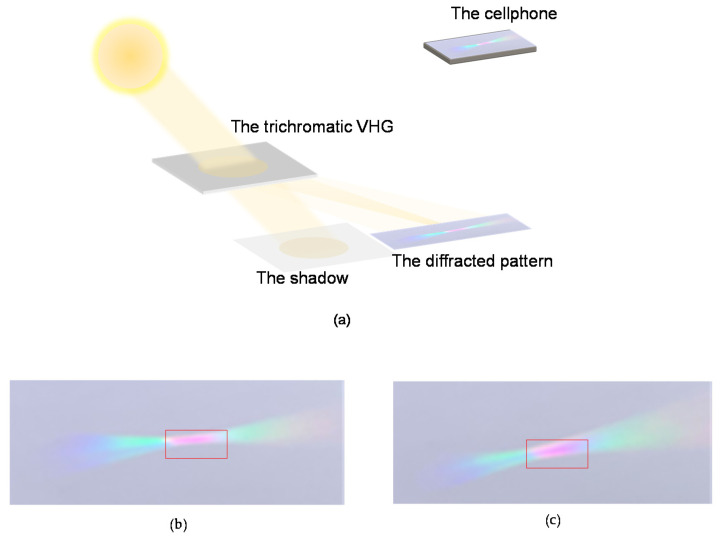
(**a**) Schematic diagram of the samples illuminated by sunlight. Light distribution on the focal plane of the trichromatic volume-holographic grating illuminated by sunlight under (**b**) the Bragg condition and (**c**) a small deviation from the Bragg condition.

We place the trichromatic volume-holographic grating under sunlight to test its convergence performance. The trichromatic volume-holographic grating is rotated so that we can determine the Bragg condition by observing the light distributions on the focal plane, as shown in Figure 7a,b. Under the Bragg condition, the diffracted light spot is the smallest and brightest, as shown in Figure 7a. The convergent white light spot shown in Figure 7a is approximately a rectangle whose length and width are approximately 5.7 and 1.5 mm, respectively. Then, the geometric concentration ratio under sunlight can be calculated using the ratio of the focal-spot area to the incident-beam area, i.e., ${Ρ}^{\prime }=S_{0}/\;S_{1}^{\prime }=27.2$. The average light energy utilization rate of the condenser under the sunlight measured by using an optical power meter (Newport No.840-C) is 21.01%.

**Figure 8:**
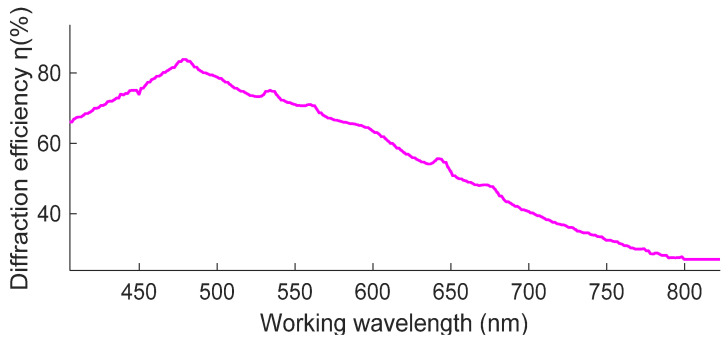
Relationship between the diffraction efficiency and working wavelength of the trichromatic volume-holographic grating.

We characterise the working wavelength range by measuring the transmission rate of the trichromatic volume-holographic grating using a spectrophotometer (Hitachi U-4100). The result is shown in Figure 8.

Figure 8 shows that the single-layer cohesive RGB volume-holographic grating has an effective spectral range of 400–700 nm, which covers most of the visible-light band. The maximum value of the diffraction efficiency in the visible band is approximately 500 nm. When the wavelength is more than 500 nm, the diffraction efficiency gradually decreases with the increase in the wavelength, and the diffraction efficiency in the visible band exhibits quite high diffraction efficiency.

### 4.4. Discussion

Compared with previous work, it can be seen from Table 1 that the proposed trichromatic volume holographic grating has the highest concentration ratio and relatively high light energy utilization rate. Although the trichromatic volume-holographic grating can reproduce sunlight, the result is not perfect because the light spot is quite red. There are still diffraction spots when one color light illuminates the different color holographic gratings. And the diffraction efficiency of the trichromatic holographic grating is larger under the red-light illumination than that under blue and green light illumination. These lead the result shown in Figure 7, i.e., the central spot is reddish and there are diffraction spots with less efficiency around the central spot. This phenomenon may be caused by the mutual interference between the three-color monomer molecules in the photopolymer material. Therefore, in the future, if photopolymer materials are used to record the volume-holographic gratings for sunlight concentration, the three-color monomer molecules in the polymer should be optimised so that the wide-band volume-holographic condensing system becomes more effective in reproducing sunlight. Furthermore, a multi-layer concentrating system can be constructed according to our present work to obtain large working-angle solar concentrators.

## 5. Conclusions

To solve the problem of high cost in the current conventional solar-concentration technology due to the tracking system, we propose a new wide-band solar-concentration technology based on volume holography. We use the Kogelnik coupled-wave theory and a photopolymer monomer diffusion model to investigate the diffraction principle of volume holography and obtain the main factors that affect its diffraction efficiency, namely, spatial frequency, exposure intensity, polymerisation coefficient, and diffusion time constant. Through theoretical analysis and calculation of the diffraction efficiency of a trichromatic volume-holographic grating, a laser-interference optical path with centre wavelengths of 473, 532, and 639 nm was constructed to fabricate a wide-band volume-holographic grating. The samples were recorded under the following best recording conditions: the exposure intensity under uniform light intensity is 0.2 mW/cm^2^, the spatial frequency is 820 lp/mm by adjusting the angle of the two light paths, and the dark reaction time is 5 min. The experimental results reveal that the single-layer trichromatic holographic grating under the illumination of the three recording lasers exhibits a concentration ratio of 149.2, a maximum diffraction efficiency of 55.7%, a working angle of 6.7°, and a light-energy utilisation rate of 26.54% within the working angle range. When the grating is under sunlight, the concentration ratio is 27.2, which is also quite high, and a light-energy utilisation rate of 21.01%. The working band is visible light. This work provides an idea for a plane concentration system, and the large-angle and wide-band characteristics reduce the cost of solar-energy collection.

## Figures and Tables

**Table 1:** Light energy utilization and concentration ratio of different structures.

Structure	Light Energy Utilization(%)	Concentration Ratio
by Bianco et al. [3]	13.83	5.85
by Lee et al. [18]	18.22	15
by Castro et al. [6]	50	1.5
by Akbari et al. [11]	18.64	20
the proposed trichromatic volume holographic grating	21.01	27.2
